# The Hospital Nursing Supplement

**Published:** 1893-08-05

**Authors:** 


### "The Hospital" Nursing Mirror.

Being the Extra Nursing Supplement of "The Hospital" Newspaper.

[Contributions for this Supplement should be addressed to the Editor, The Hospital, 428, Strand, London, W.C., and should have the word "Nursing" plainly written in left-hand top corner of the envelope.]

### News from the Nursing World.

### INCOMPETENT TO CRITICISE.

'Tis an old saying amongst hospital nurses that the newest probationer generally constitutes herself the chief critic on ward management, and also on institutional administration. To such we cordially commend a saying of Miss Isabel Hampton's, "In her first month in a hospital the beginner will probably only succeed in becoming familiar with her surroundings, a few nursing appliances, and some of the minor steps in nursing."

### INCREASED ACCOMMODATION FOR NURSES.

The foundation-stone of the new buildings was laid last week at the North Western Hospital, Haverstock Hill. The increased accommodation will give ample space for the nursing staff, who will be provided with new dining, sitting, and bed rooms. There is to be a famous kitchen for the preparation of all the food used in the hospital, and also offices for the medical staff. When this block of buildings is completed, plans for the rebuilding of the wards will be carried out, and these will enable the hospital to accommodate twice its present number of patients.

### MERITS AND DEMERITS.

Out-door uniform for nurses is getting attacked and defended with equal zeal just now, and the reasons given for and against it are many and varied. Surely it is just one of those professional distinctions of which the use is as admirable as the abuse is disastrous. There is an old saying which might well be applied to the wearers who are bringing discredit on their calling by personal vanity or indifference in the matter of dress: "Let each one mend one, and let that one be herself!"

### FOR PATIENTS OR NURSES?

Again and again thoughtful persons are driven to inquire, for whom do hospitals now exist? It was formerly understood that they were dedicated to the service of the sick poor, but the latter have been rather relegated to the background by the public at large. Whether or not a hospital suits its nurses is now apt to be the first inquiry made. Hence has arisen the belief, too frequently held by many modern nurses, that sick people exist for their benefit, instead of the reverse being the case. It is to be hoped that more reasonable views will be eventually entertained, and then the "servants of the sick" will assuredly receive honour, even as they themselves bestow it on the patient poor.

### NO FRESH AIR!

Most nurses are ready to own that they don't go out enough, and that it is unfortunately very easy to slip into the habit of staying in which seems to them perchance a strictly personal indulgence. But it is not so, for a nurse's general "fitness" is of moment to all with whom her duty brings her into contact—and this "fitness" cannot be secured without reasonable relaxation and fresh air. Yet the recent statement of a contemporary is a somewhat extreme one, and we read it with grave surprise. "Very often, indeed, a nurse never so much as gets a single breath of fresh air for weeks together." Certainly, if such be the case this offender against the laws of health should be advised to make better use of the daily hours off duty which are rightly her own. Such sweeping statements are read with surprise and amusement by the experienced nurse.

### WHOSE FAULT IS IT?

A man was summoned last week for allowing his two children, who were suffering from scarlet fever, to mix with others and also to attend school. The offence was not committed through ignorance, for the medical officer had previously warned the father and mother on the subject. Judging from the report in the papers there seems no possible excuse for this unpardonable behaviour, and the wilful ignorance, to call it by no harsher name, which exposes both neighbours and school fellows to the risk of an epidemic of scarlet fever, should be preventable. Surely there must be some imperfection in a Public Health Act which allows loopholes for such disastrous proceedings.

### VALUABLE LECTURES.

The Council of Queen Victoria's Jubilee Institute provides lectures for the probationers at the Metropolitan National Nursing Association, where training in district work is given to those who wish to qualify as Queen's Nurses. Dr. Cheadle and Dr. Sidney Coupland have given lectures on hygiene, and Mr. Warrington Haward on anatomy, and Mrs. Scarlieb, M.D., a course on the diseases of women. This valuable instruction is highly appreciated by those privileged to share it.

### UNBUSINESS-LIKE CORRESPONDENTS.

The superiority of the male sex in any way whatever is seldom acknowledged by the women of modern times. Equality may be granted to them, perhaps, but certainly nothing more. Nurses are specially tempted to think themselves on a higher elevation than the hapless sick man, who is often as helpless and as dependent as a child on their care and skill. Yet our nurses might well be content to copy the business habits which past centuries have made so much easier to men than to the slowly developing and educated woman worker. Would any man, we wonder, send in contributions without signature, and when he discovers his omission, would he send his name and address by a second post without any explanation of what it belongs to? And would he complete the series by inditing a third letter telling just the simple facts, which would have made all clear in the first instance, and thereby have saved time and postage stamps. Many people can adequately sympathise with the annoyance which attends the receipt of these unbusiness-like communications.

### RURAL NURSING IN LINCOLNSHIRE.

A new County Rural District Nursing Association was lately inaugurated at Lincoln at a meeting at the Chapter House, at which the Dean presided, supported by the Earl and Countess of Winchelsea. It was resolved to seek affiliation with the Q.V.J.I., which is sufficient guarantee that the Association will aim at supplying only properly trained and qualified nurses for the sick poor in their own homes. The Countess of Winchelsea was elected president, and the Hon. Lady Thorold and the Hon. Lady Stanhope vice-presidents, £120 was promised at the meeting towards the £200 required for the initial expenses.

### PROGRESS AT BANGOR.

The Ladies' Committee seems likely to be of practical value in the administration of the nursing department at the Bangor and Beaumaris Union. The members not only themselves realise the absolute necessity for the engagement of a trained nurse, but they are endeavouring to impress this need upon the Board of Guardians. The difficulty of getting a fully-qualified nurse who speaks the Welsh language is somewhat of a drawback to the scheme. It is, however, a difficulty which ought now soon to disappear, for there must be plenty of intelligent Welshwomen ready to go through a regular course of training, and so to fit themselves to follow the useful and honourable calling of charge nurse in a workhouse infirmary.

### NEW YORK.

The notices of the annual commencement of the New York City Training School for Nurses were issued last month, printed at the New York City Asylum for the Insane at Ward's Island. The invitations were issued for July 6th by the Commissioners of the Department of Public Charities and Correction, and the usual business of the meeting was transacted in a most satisfactory manner.

### AN AMERICAN NOVELTY.

The Emergency and Hygienic Association at Massachusetts has recently undertaken to train attendants to supplement fully-qualified nurses. It proposes to instruct young women systematically, and to fit them for taking charge of chronic invalids, aged persons, convalescents, and delicate children. They are intended to succeed trained nurses in cases where the latters' services can be dispensed with, and yet proper care is still necessary, The plan sounds well enough, but would need very serious consideration before being generally adopted. Although aged persons and convalescents would often do quite well with attendants of average intelligence, yet sickly children certainly need not only careful but experienced treatment, and nothing less will give them much chance of developing into healthy men and women. The instruction probably will be valued and valuable, but necessarily limited. An accurate definition of the position which these supplementary attendants can safely aspire to hold should assuredly be enforced for the protection of their patients and the public generally.

### A NEW HOME AT VANCOUVER.

A home for medical and surgical patients has been recently opened at Vancouver by an English trained nurse. It is to be a self-supporting nurses' institute, and as such should deserve success. Miss Wilkinson, who has established it, was trained at the London Hospital, and was afterwards for five years at the Gloucester County Infirmary; and Miss Woodward, a devoted and excellent nurse, who is associated with her, was trained at the City Hospital, Vancouver.

### ENGLISH NURSES IN PARIS.

The rapidity with which the journey to Paris can now be accomplished, makes that capital appear very near to us, and yet we seem to know but little of the work of our French sisters. We hear from a correspondent that an institution for fully trained English nurses conducted on sound co-operative principles, would be likely to succeed in Paris; but those who contemplate any such undertaking would do well to acquaint themselves with the amount of demand for English women which exists in France, and in fact make sure that nurses going over would get regular employment.

### INDIAN NURSING.

The Ramsay Hospital at Naini Tal is beautifully situated, and the patients recline at their ease in the broad and lofty verandah, from whence they have a magnificent view. The building itself is very fine, and from its handsome portico a vast expanse of country meets the eye, with endless variety of colours, of lights and shades, of mountain and lake. Miss MacVitrie recently resigned her position as lady superintendent of the Ramsay Hospital, and has been succeeded by Miss A. S. Wildman, a sister of Miss E. A. Wildman, who is superintendent of lady nurses at the Station Hospital, Umballa. Patients seem to be made very comfortable, and the monotony of sickness appears to be lessened as much as possible. Certainly skilled nursing amidst such admirable surroundings must accomplish many excellent cures. On the side of the hill above the Central European Hospital there are two long piles of buildings, of which one is reserved for male and the other for female Hindu and Mahommedan patients. The houses of the lady superintendent and the resident surgeon are also detached buildings, and the arrangements of all the departments are described as excellent.

### SHORT ITEMS.

At a bazaar recently held at Lochwinnoch on behalf of the local nursing society £318 7s. 6d., was collected during three days. This included £12 16s. 6d., the proceeds of an amateur dramatic entertainment. The society employs a Queen's Nurse.—Dr. E. Liddon presented the prizes to members of the Nursing Institute at Taunton the other day.—The committee of the Clapham Maternity Hospital are appealing for a sum of £2,500 to purchase the freehold and also to enlarge the hospital in Jeffrey's Road. Subscriptions to this fund have been already received from the Worshipful Company of Clothworkers, £10; the Grocers' Company, £25; the Cordwainers, £21; and the Skinners, £10 10s. —The Elham Board of Guardians have set a good example in deciding to advertise for a certificated nurse at a salary of £35, instead of £25 as previously suggested.—The nurses at the Emergency Hospital at the World's Fair are under the charge of Miss M. R. Brown, a graduate of St. Luke's Training School, Chicago.

### THE DISABLED NURSE.

Our readers will be sorry to learn that the disabled nurse, in whose case so many of them have taken a kindly interest, is very ill just now. A friend, who is also a nurse, has written in her name to thank all those who have helped her. The subscription list is now closed; the last instalment of the sum of £14 12s. 6d. collected was forwarded to the invalid last week.

### Sanitation for Nurses.

By a Sanitary Inspector.

### XVI.—METHODS OF DISINFECTION IN FOREIGN COUNTRIES.

In France the responsible duties connected with disinfection are entrusted to the Prefecture of Police. Rooms are disinfected by sulphurous acid gas, produced by burning sulphur, 20 grammes being allowed for each cubic metre.

Most of the hospitals have apparatus for disinfecting by steam, and lately the Municipal Council has approved a scheme for regulating disinfection, and for building two disinfecting stations.

The stoves are to be fixed into the wall between the two chambers destined respectively to receive infected and disinfected articles, and the stoves are to be arranged with an opening at each end for the same purpose. The stations are very complete, with a house for the superintendent, so arranged that he can see both ends of the stove from his windows. The stations are to include a bath-room for the disinfector's ablutions, and also a laundry. The staff is to be composed of no fewer than ten persons, who are all to be lodged and boarded at the station. In the case of infectious disease breaking out in a private house, as soon as the commissioner for that particular quarter is informed he sends to the family, and offers free disinfection by the public disinfector, and if agreed to the rooms are fumigated. There appears to be no provision made in case the family decline the trouble and inconvenience of being fumigated, and one can only suppose that if the offer be declined this most necessary precaution is omitted. The stove most generally used in France for disinfecting is manufactured by Geneste and Herscher.

In Brussels we also find sulphurous acid gas used universally and exclusively for houses as well as clothing and other articles. All disinfection has to be carried out in the houses in which the infectious disease has broken out. In the case of bedding and pillows, these are removed, and the contents exposed to sulphur fumes, whilst all articles of small value are burned. For disinfecting excreta and drains a solution of copper sulphate or of weak carbolic of only two to three per cent. strength is used. Linen is disinfected by boiling in a solution of zinc chloride or of zinc sulphate and common salt.

In Germany, as one would expect, the regulations regarding disinfection are very minute. As regards the disease after which the room occupied must be thoroughly disinfected, the rules are that—after cholera, small-pox, typhus, remittent fever, and diphtheria the occupants are bound to disinfect; but in cases of typhoid, "malignant" scarlet fever, or dysentery, the disinfection is to be done "if required by the police"; whilst after measles, whooping-cough, and pulmonary phthisis disinfection is compulsory. The distinction made between these last two groups of disease are curious; and why in the case of the last three disinfection is compulsory, but after "malignant scarlet fever" the need for disinfection is to be left to the judgment of the police, of all people, it is very hard to see. The public are carefully informed by what means infection is carried in general, and in cases of special diseases whether by excreta, vomit, &c., and some useful rules are laid down for the care of the patient and the sick room, although it is stated that "the patient should be washed every day"—an excellent rule no doubt, but one would have imagined it would have been better to leave it to the discretion of the doctor and the trained nurse, as some over-conscientious persons, anxious to carry out the rules implicitly, might show more zeal than discretion on this point. Steam at high pressure is used, but all the same, directions are given for boiling linen for half an hour, and afterwards for washing in a solution of about 5 per cent. carbolic acid. It does not appear that fumigation is used in rooms. The orders are as follows: "At the close of an illness all articles which cannot be washed, such as bedding, silk, carpets, furs, stuffed furniture (excepting the wood), are taken away with care, without beating or shaking, to the disinfecting establishment, after they have been wrapped in a cloth soaked in a solution of carbolic acid. Leather is washed with the solution. Articles of small value are burnt in a stove belonging to the house. Cooking must not be carried on at the same time. Large articles are burnt in the disinfecting establishment. Polished furniture, pictures, objects of art, metals, &c., are rubbed with cloths; bread is used for the walls and hangings. The floor must be first wetted with a strong solution of carbolic acid. If the walls have been soiled with the evacuations, they must be wetted with the carbolic solution and then scraped. All floors, without exception, doors, windows, and woodwork not polished, must be washed with a strong solution of carbolic acid after cases of cholera, small-pox, diphtheria, spotted typhus, remittent fever. The solution must be injected into the crevices of the parquet. Everything must afterwards be washed with pure water. The bread and cloths used in cleaning must be thrown into the fire. Any cloths to be used again are soaked in a weak solution of carbolic acid for twenty-four hours, then boiled and washed with potash soap. After disinfection the room must be aired for twenty-four hours." Disinfection is entrusted to a specially trained person, who superintends and is responsible, and the disinfectors who work under him have to be approved by the administration. The disinfectors have to wear a special dress whilst engaged in their work, and afterwards are required to wash thoroughly and change their clothes.

The stoves used for disinfection are very similar to those used in France, and Berlin possesses two disinfecting stations, each provided with three stoves.

In Austria the universal disinfectant is carbolic acid, whilst sulphurous acid gas is used for disinfecting rooms. A 5 per cent. solution is used for washing furniture, walls, and floors, and all linen which will wash is first steeped in carbolic of the same strength for twenty-four hours, and then washed in the usual way. Disinfection by steam is used, and several kinds of apparatus are in common use in Austria for this purpose, though the one most commonly employed is one constructed by Thursfield. In Vienna there is a disinfecting apparatus in each "arrondissement." All bacteria spores are said to be destroyed on exposure for half an hour to steam, and hence that is the time occupied by each process of disinfection.

In Sweden and Stockholm the methods of disinfection are very similar. In Sweden disinfection is done at a public station, under the care of an inspector, whilst houses are fumigated in the usual way with sulphurous acid gas, and are afterwards very thoroughly cleansed and ventilated.

In Finland the same arrangements are found as in Paris. The disinfecting stove is made on the same principles as those of Geneste and Herscher, and the arrangements at the station are similar. The "Health Police" undertake the disinfection of private houses under the direction of the city doctor, but there is no compulsory disinfection for any infectious disease at present.

The most striking point with regard to disinfectants in foreign countries is the want of variety in those used. The list of those which find favour is very short. Sulphurous acid gas and carbolic are exclusively used except in France, where sulphate of iron and chloride and sulphate of zinc are occasionally used. In any case there is nothing at all like the variety of disinfectants used in this country. Corrosive sublimate finds no favour at all. It was tried for a time in Germany, but was condemned as unsatisfactory by Koch, who was the first to bring it into disfavour by declaring it to be an inefficient disinfectant of fæces, and other authorities in Germany declared it did not destroy tubercular bacilli. Hence, though it was included in the fresh regulations made in 1883, it was afterwards excluded in the later regulations of 1887, on the authority of Koch, and also on account of its very poisonous nature.

### District Nursing.

### THE PARISH NURSE.

Sick nursing among the poor is, at the present time, awakening a good deal of attention. The lower classes in towns and villages are slow to adopt newer principles of sanitation, and it is necessary to wean them gradually to habits of cleanliness. Feeling an interest in the work, a friend of mine began in a small country town. The place was not densely populated, neither was it an idle one, for there were two or three factories, which gave employment to many hundreds of people.

We were advised, for we had determined to set up house together, to become the tenants of a cavernous mansion, well supplied with cellars, and warranted genteel. However, we preferred a much humbler abode, overlooking the borough lands, where the wind rushes in from the sea. This is situated in one of the centres of the poorer folk, and, therefore, well suited for our purpose.

My friend the nurse had been trained in a hospital for several years. Finding, however, as time went by, that the ward work was too much for her strength, [she turned her thoughts to this branch of nursing. Though undeniably fatiguing, it offers more variety and demands less of particular muscles and nerves than the other, and to a good walker, resembles more nearly a normal and healthy country life.

She goes out every morning at about ten o'clock, to make her first round, pretty much like a doctor, spending ten minutes or a quarter of an hour in each house. Helpless and lonely patients may detain her much longer, but if there are relations and friends her duty is to direct more than to undertake the actual nursing. One of the gravest difficulties in nursing, generally, is to control the patient's relatives, and prevent their interfering with the ministrations of the nurse. A sensible and well-trained helper among the poor will be able to overcome this, but she must have material aid at her back. Granted the possession of tact, added to the nursing faculty, it is still impossible to do without a few beds at home for cases requiring constant attention. How can a nurse go her rounds, perhaps to twenty patients, and at the same time control the movements of some restless invalid, whose only hope of health in the future depends on her lying perfectly still? Her relations are busy, or they are too lazy and ignorant to apprehend the danger, or to attend to her properly, so the only safe plan is to remove her for a short while.

A good "sick kitchen" whence nourishing food can be sent out, well cooked, and suitable to the exact needs of each patient, is a necessity. For a woman slowly recovering from a serious illness, or a consumptive with a failing appetite, a black and greasy chop is not an inviting dinner, nor the pudding falsely called "milk"—dry and hard. Failing a regular "kitchen" however, much may be done at home. Nurse is allowed a small expenditure monthly for the needs of the sick, and this is laid out carefully on fresh, juicy meat, for beef tea or mutton broth, or for convalescents a chop gently stewed (with vegetables if allowed), and sent out hot and appetising just at dinner-time. Jellies, with or without wine, custards and blanc-mange can all be made with a little thought for a very small sum.

Next winter we hope to get up a proper system of regular hot dinners for the old and ailing, good solid food, two or three times a week. As Nurse says, "What is the use of poulticing bronchitis and bad legs when it is the system that is at fault?" To make two and sixpence a-week hold out when in health must be a problem, with rent to pay and firing dear. But a little charing or cleaning, or even knitting, brings in a trifle. Think of it! when the old hands drop down, and the kitchen fire is out, and the bed-room grate holds but a mere morsel of coal and is much given to smoking. Then nothing comes in but woe and want, parish bread, and a block of fat and bony meat; the "house" looms in the distance, and heart and body sick and depressed re-act upon each other. One of the most curious features in the life of the poor, is, that the old and forlorn women so rarely coalesce, and live together, sharing rent, lights, and firing, with the added advantage of being able to help each other in their hour of need. They seem to think, as the old North countrywoman phrased it, "I maun hae me own fireside til mesel, wer't only to take the pet by."

Another part of nurse's duty, and this needs great care and tact, is to get the rooms and houses washed down and scoured out after an illness is over. In cases of infectious disease, she gives a sulphur candle to fumigate beds and bedding, but as she is not allowed to visit these cases, she can only advise, or lay the matter before the Sanitary Inspector. During the illness, also, the use of soap and water has to be enforced and some compliance is generally attained, in deference to the "lady's" wish. There is, indeed, one decided advantage in having a lady nurse, *i.e.*, such an one, as the poor, none better, can easily recognise. What they would not put up with from one of their own sort, or of a little higher rank, "pokin' round" (as Aunt Dinah would say), they take meekly enough as a harmless peculiarity of the lady nurse. "Well, there, it ain't fit for the likes of her to sit her down in sich a dirty place," they say, considerately; and in charitable deference to the lady's weakness, more than any real sense of the necessity of the case, the filthy floor is scrubbed and the bedding replaced by willing neighbours; clean sheets are lent by the nurse, and a little light and air allowed to creep into the stuffy bed-room.

In this way a moderate-sized town can be well worked and efficiently guided into paths of cleanliness and sanitation in a manner beyond the reach of mere technical education classes. These invite the people to come and learn from lecturers, whose terms and language even are abstruse to them; nurse invites herself, and teaches them by the simplest demonstration lessons. It is a power for good this nursing system, the like of which has not been seen, except perhaps in the Zenana. Where ignorance and vice are rampant the nurse goes unchecked and unharmed, and hard must be the heart and brutal the intelligence which learns nothing at all from her gentle tending.

The nurse is also a channel of benevolence. From many who abhor indiscriminate almsgiving there flows a steady stream of help to those who, bad or good, are in dire need and distress. She knows exactly what they want and what they do not want; measures the brandy by the spoonful, and possibly sees the patient drink it; and can tell precisely where blankets, food, and fire will be a real boon and comfort. Let us hope that not many years will go by before every hamlet has, together with parson and doctor, there natural complement, the parish nurse.

### Wants and Workers.

Nurse E. will be very grateful for information as to prospects for a private English nurse starting on her own account in Montreal.

Nurse S. wants to know of a home for an idiot boy nine years old. A small sum of money could be paid towards his maintenance.

### Our Examination Questions.

Some of the answers to the July question are particularly good, and all of them go to prove the writers careful and intelligent bed-makers. It is true, as most competitors say, that it is always best to have the assistance of two persons when the patient is very ill or extremely helpless, but it is pleasant to find that one at least has described a method in which a careful nurse can manage to make a bed single handed. Another, with rather luxurious notions, thinks that three are needed, but it is in very exceptional cases that so many people are either necessary or desirable. Only one writer mentions the excellent plan of placing a footwarmer under the patients' feet when he is first allowed to get up whilst his bed is made. It is often convenient to arrange that the tin shall be refilled and placed in readiness before the invalid is disturbed.

Question for July.

A. Describe carefully the different ways of making a bed with a patient in it. B. The precautions to be taken when a patient gets up whilst the bed is made.

Prize Answer.

A. The beds of helpless patients, *i.e.*, those not permitted to leave their beds for the purpose of making, may be done by the following methods: In the case of a patient entirely prostrate two nurses or bed-makers are indispensable. The upper bedclothes being removed with the exception of a light covering, preferably a blanket, the patient should be gently turned on his side and supported in that position by one attendant, while the other smoothes out the under sheet, mackintosh, and blanket, if one is in use, sweeps out any crumbs, &c., and places the draw-sheet so as to bring a fresh piece under the patient. He is then turned carefully on to the opposite side, and the second attendant proceeds in the same manner as the first. The patient is then turned on his back, and one supports the head, while the other removes the pillows from the bed and thoroughly shakes and replaces them. The upper bedclothes are then put on, turning the upper end of the sheet over the counterpane and blanket to a comfortable height round the patient's shoulders. For a patient who is obliged to sit up, as in many cases of heart disease, the same plan may be followed out from top to bottom of the bed, instead of from side to side. When the sheets have to be changed in either case, the soiled under one should be rolled up half way, close against the patient's back, the half of the clean one also rolled up and placed against the soiled one; the patient then turned in the one case, or lifted in the other, and the soiled one removed, while the clean one is unrolled and straightened out. The draw sheet should be changed at the same time, and in the same manner, so as to avoid turning or rolling the patient more than once.

B. For a patient getting out to have the bed made the following precautions should be taken: (1) He should be warmly wrapped up to avoid a chill, particular attention being paid to the feet; if necessary a hot-water tin should be placed on the floor to put them on. (2) He should be carefully sheltered from draughts. (3) He should not be out of bed longer than is absolutely necessary.

B. Austin.

### Novelties for Nurses.

Messrs. Garrould's stock of nurses' uniforms includes every detail of a complete outfit. Bonnets at moderate prices, with or without veils; caps of many kinds, besides the admirable "Sister Dora" shape, which is sold for a shilling by Messrs. Garrould in their Edgware Road establishment. Collars and cuffs, aprons and ward shoes, and all kinds of dress materials are on view. A probationer can select what she requires, and get measured for her gowns, and can then dismiss the subject from her mind until the date arrives for her to appear in uniform. Messrs. Garrould have a large stock of nurses' cloaks suitable to all seasons of the year, and they have some delightful cheap serge costumes which are certainly most useful for wearing during the seaside or country holiday, for which so many are on the eve of departure.

### Lady Nurses for Officers in India.

By One Who Knows Them.

The Hospital gave vent to a little exclamation of satisfaction on this subject a few weeks ago. Since then we have heard another side of the picture. Said an officer to us: "The idea of lady nurses is a delightful one, especially to any man who happens ever to have been nursed by a military hospital orderly only. One imagines the soft voice with un-Atkins-like accent, the gentle touch, the soft footfall, and the kindly intuition which forestalls one's wants before one utters them. But military stations in India are crowded with men out of all proportion to the number of women, so the nurses can have a 'good time' socially. Now, don't think me a brute to grudge them this. Nursing must be tedious work, especially nursing short-tempered fellows in a trying climate, and the nurses must need relaxation more than any of us. But I can assure you, from personal experience, that though the lady nurses even surpass one's hopes by their management of patient and sick room, still it is anything but soothing to a sick man to have a charming woman to perform all manner of unpleasant jobs for him when hitherto the onus has been on him when meeting her constantly like any other lady in the society of the place. He, like the other men of the station, has been ever 'politeful' to her as to the other ladies, not allowing her to reach for a chair or set down a teacup for herself. Judge then of a man's feelings when he has to lie still and see her perform many distasteful things for him, feeling bound to be on the alert to thank her for each kindly office, and determining to make as few demands as he possibly can. A sick man ought to be able to feel at ease instead of worried. To get well he ought to be allowed to be a bit selfish, even to feel intensely grateful in the aggregate, but not specifically for each office performed. Above all, a man lying helpless ought to feel able to ask freely for what he wants or fancies. To suppress the desire to ask for this or that to be done for one, just because one hates to give a lady trouble, and because one is already so grateful for her care and kindness, is not a health-restoring frame of mind; that way feverish symptoms lurk. A man wants to feel towards his nurse much as the French towards the *sœurs de charité*. Many of them are young and as lovely as loveable, but they come like ministering angels just while one is ill, and when one recovers they seem to vanish, as the angels might, to some hallowed spot, living apart from the busy world, which, with its mercenary cares and ambitions, they have voluntarily renounced that they may devote themselves entirely to the sorrowful and suffering only. I don't mean we want nuns in India, and I don't mean I grudge the lady nurses any fun that comes in their way. I have no plan of reform to suggest, I only tell you the result of my experience, so that if you are interested, as you say, in the subject, you can discuss the matter with the competent authorities.

### Queen's Nurses.

Queen Victoria's Jubilee Institute for Nurses (Scottish Branch).—Intimation has been received that the names of the following nurses, on the satisfactory completion of their training, have been submitted to her Majesty and placed on the roll of Queen's nurses: Lilian Hall, Lochwinnoch; Madge Rutherford, Blairgowrie; Agnes Tait, Buckie; Sarah Buchanan and Anne E. Hughes, Glasgow; May Colvin, Paisley; Isabella M'Dougall, Elizabeth Birrell, and Jessie R. Mitchell, Dundee; Ellen E. Wilson, Peebles; Emily J. Hill, Berwick-on-Tweed; Amelia M. White, Galashiels; Margaret Junner, Anstruther; Margaret Brown, Kirriemuir; Margaret M'Nab, Castle-Douglas; Nora M. Musson, Dumfries; Janet M. Paton, Kilmarnock.

### Everybody's Opinion.

*[Correspondence on all subjects is invited, but we cannot in any way be responsible for the opinions expressed by our correspondents. No communications can be entertained if the name and address of the correspondent is not given, or unless one side of the paper only be written on.]*

### THE CAPE MISSION.

Nurse Edith writes: Can anybody give me any information regarding the Cape Mission? I am anxious to go to Johannesburg as a private nurse, and would like to know if it is necessary to be fully trained and certificated to become a member of that Mission? I can obtain good testimonials of character and efficiency from matrons and doctors, but no certificate is granted in our infirmary. Would a private nurse be likely to succeed out there on her own account? I shall be truly grateful if space to answer can be spared.

### CONDY'S FLUID.

H. J. B. Condy writes: Our attention has just been directed to an article which appeared in The Hospital Nursing Supplement of 15th inst. under the heading "Sanitation for Nurses," will you kindly allow us a little space to correct an erroneous and misleading statement which it contains. In the paragraph to which we refer it is distinctly inferred that permanganate of potash and Condy's Fluid are one and the same thing. The potassic salt, which is comparatively poor in oxygen, is not one of the ingredients of Condy's Fluid, as anyone may ascertain by evaporating a little of our preparation and examining the residue. The similarity in colour between solutions of the different permanganates has led many persons into the same error.

⁂ Our contributor writes:—It was no doubt inaccurate to say that permanganate of potash is the salt used, whereas in reality it is permanganate of sodium. As, however, these salts are so largely interchargeable, though sodium is most generally used on account of its greater cheapness, the mistake does not appear to us to be a serious one. Moreover, in text books Condy's Fluid is frequently put in parenthesis in speaking of permanganate of potash. From whichever salt derived the remarks in "Sanitation for Nurses" regarding the use of Condy's Fluid as a disinfectant, appear to us to hold good.

### THE LONDON HOSPITAL.

Nurse Martin writes: When I saw the account of the *Pall Mall Gazette* of the London Hospital I thought you would not mind my writing a letter to the "Nursing Mirror" regarding my treatment as a probationer from 1881 to 1883. When I first entered as a probationer we had our breakfast and tea in the wards. On Saturday night we each had a mutton chop given to us for our Sunday dinner in the wards, to be cooked by ourselves if we could find time; if not, we went without our dinner. But for the great fight which the present Matron had we should not have had the great change both for the general comfort of patients and general nursing staff. Her one thought was to do all that was in human power to improve the general comfort of the hospital. I can truly say that I look back to my two years' training as the happiest two years of my life. I cannot speak too highly of the Matron, Miss Lückes, and I feel proud to be able to say that I have been trained under her. I cannot say how very sorry I feel that one of our best and oldest hospitals should be so very unfairly treated by those people who very often enter under the cloak of love for the work. Very often it is because girls cannot get on at home, and cannot find a better excuse to get away, or very often out of pure love of change. I am quite sure that the grand work which has and is being done there must sooner or later win the general support of the public. If you will kindly allow this to be put in the "Nursing Mirror" I shall feel greatly obliged.

### OUT-DOOR UNIFORM.

"Nurse C" writes: In reply to "An Old-fashioned Nurse" I beg to explain that out-of-door uniforms save both time and expense, and for that reason—not for love of singularity or parade—we value them. They save time because instead of changing our dresses and arranging our hair so as not to look "singular," we can just put on our cloak over our uniform, and our bonnet over our hair (arranged high to suit our caps), and so save ten minutes or so at each end of our walk. They save expense because uniforms do not change fashion, or need not, they do not involve expensive boots and gloves, and all the little extras necessary for the finish of a correct toilet. Between a heavy lined cloth winter cloak, and a light summer one, there is difference enough to satisfy nurses in general. How a cloak and bonnet can be a costume "ignoring health, comfort, and economy," I fail to comprehend. I find a uniform particularly comfortable and walk miles in it when off duty, and does "militarism" in soldiers, "priestcraft" in ministers, "hold these 'servants' unnecessarily apart from those for whose service they exist"? Because a nurse is before all things a nurse and takes all means (even an out-of-door uniform) to economize the time and thought she desires to devote to her work, is she likely to be less capable or womanly than one who spares time and thought for dresses suitable for "riding, rowing, tennis, and woodland rambles?" The "one thing needful" is to be really what we appear—priest, soldier, or nurse—let us neither "drift" nor be "driven," but walk right on towards that end, for concentration, enthusiasm, and thoroughness are good and not evil—even in out-of-door uniform.

### ANOTHER OPINION.

"A Country Nurse" writes: Not many women, I venture to say, will agree with "An Old-fashioned Nurse" that outdoor uniform is only necessary to district nurses. Outdoor uniform is a great boon to many nurses where the pocket is concerned. A woman with common sense engaged in nursing the sick would abominate excess of any kind in her dress, especially noisy chatelaines and jewellery, but unless she has a private income she is very glad to adopt the neat out-door garb that can be slipped on so easily and quickly. The veil at the back is not necessary. Naturally every woman likes to dress becomingly, and an ordinary bonnet soon gets dowdy, unfashionable, and unbecoming, whereas one feels perfectly comfortable in shabby uniform providing it is neat and quiet. In regard to riding and rowing, a nurse's work is too absorbing and her recreation time too limited for her often to indulge in those pleasures, and if during her short holidays she can afford costumes for them, that alters the case. The majority of nurses I have met have only their earnings to dress upon.

### Minor Appointments.

General Hospital, Tunbridge Wells.—Miss Ellen Nott has been appointed Sister of the children's ward at this hospital. Miss Nott was trained at the Royal Albert Hospital and Eye Infirmary, Devonport, where she was subsequently Staff Nurse and Acting Sister.

Hyde, Cheshire.—Miss A. Annette Tinkes has been appointed district nurse to the town of Hyde, Cheshire. She was trained at King's College Hospital, 1882-83; since then for upwards of 10 years has been engaged in district and private nursing for the Bromhead Institute, Lincoln, where she was much esteemed by all who knew her. Many sincere good wishes follow her to her new sphere of usefulness.

### Free Lectures.

### II.—SALINE SOLUTION.

Dr. Sherrington gave his second free lecture at the London University on July 12th, and began by stating that whenever a horse died or had to be killed its blood was collected and examined with great care. Hence by repeated estimation it was found that a horse's blood was one-eighth of its whole body weight, hence a horse has more blood in proportion than a man, whose blood is about one-thirteenth of his whole body weight. It has been found that as a rule the stronger the animal the higher the percentage of blood.

Experiments in injecting normal saline solution have proved that when introduced into a vein slowly and by repeated injections, the blood pressure rises slightly, but very soon falls to nearly the original level. Even if solution be injected so as to increase the original blood-flow one-third, the added tension in the arteries is only slight, whilst the circulation does not quicken nor is there any œdema. The only change is in the lymph, in the thoracic duct, the flow of which is increased, and it has a higher percentage of solids, that is it is more concentrated. If the original amount of blood be increased to more than half, the circulation becomes embarassed and death will supervene. Up to a certain limit the circulation can adapt itself to an increase or decrease in the amount of blood. Animals, however, suffer from faintness, when, after injection of the solution, less blood is withdrawn by venesection than the normal amount.

Bright's disease is said to be due to a watery condition of the blood, &c., lower specific gravity often 1015 instead of 1020, resulting in œdema and dropsy, but even if some of the blood be withdrawn and replaced of an equal quantity of saline solution the same symptoms are not produced.

If saline solution be injected rapidly it is got rid of out of the blood of the secreting glands nearly as rapidly as it is injected, but the kidneys do not get rid of it, only the liver and intestines do their share.

### New York City Training School for Nurses.

This institution is situated at Blackwell Island. We are indebted to Miss Louise Darche, Superintendent, for the following results of the recent examinations.

Graduating Class, 1893.—Emma B. Miller, Bertram O. Gardner, Anna E. Lavelle, Carrie Gray, Grace Inez Richards, Charlotte S. Stegen, Helena Gallagher, Celia A. Knapp, Helen Adell Taber, Jeanette McMillan, Eva G. Foster, Isabel D. Montgomery, Jennie S. Doland, Florence Shoemaker, Myrtie Bell Snyder, Mary J. Wilmot, Fannie M. Morris, Josephine Hill, May Park, Elizabeth Curry, Margaret Alvaney, Mary J. Macfie, Sarah Anna Hall, Eva Saunders, Laura A. Cross, Lillian Ross, and Armavenie H. Ishkanian.

Senior Class.—Morella Starkweather, E. Blanche Edwards, Grace Watkins, Maggie Thompson, Mary Carson, Jane Bailey, Jennie L. Chidsey, Maude Keeney, Eleanor I. Hopkins, Catherine Barr, Anna W. Levering, Hattie Gorr, Margaret Armstrong, Flora Robinson, Pauline Seaman, Jennie A. Travis, Nettie M. Huey, Eliza L. Stage, Kate Tureaud, Isabel M. Bamber, Sallie S. Bell, and Lucella P. Kimble.

Junior Class.—Edith E. Wait, Jessie A. Stowers, Emma Mills, Delia Renehan, Annie Haight, Margaret G. Pierce, Mary W. Whittaker, Agnes McIntyre, Nellie K. Mobbs, Nellie C. Douglas, Mary Carroll, Minerva Shields, Nellie Wilmot, Lizzie O'Connor, Minnie Lunger, Hildah McGilvray, Mae Lounsbury, Mary Donovan, Minnie L. Coleman, May Mobbs, Florence A. Walker, and Lillian Farnham.

Officers of the School.—Diana C. Kimber, Assistant Superintendent. *Supervising Nurses.*—Irene Morgan, Delia Riggs, and Charlotte Anna Allen.

### A MATRON'S RESIGNATION.

We regret to hear that Miss S. E. Allen, in consequence of ill-health, has been obliged to resign her appointment as Matron at the City of London Lying-in Hospital, a position she has held for nearly nine years, with credit to herself and benefit to the hospital and patients under her charge. The Committee will have great difficulty in filling her place.

### For Reading to the Sick.

### ASPIRATIONS.

We often feel that from ill-health and other afflictions we are totally unable to rise above the little cares and troubles of life which tie us down and prevent our being religious, as we call it. "It is enough," say we, "to get along at all, there is no room in our lives for cultivating love to God and man." But in this we make a grievous mistake, "Where there is a will there is always a way" quoth the proverb. See what large houses they build in London on a little ground! In that enormous city land is so valuable that the builder is obliged to keep on a very narrow strip, and when he wishes to erect a large house he makes up for the narrowness of room by piling up storey upon storey, and thus, as they say in arithmetic (to quote an old writer), "Multiplying many chambers from the root of one floor." And though when thus built it is sometimes painful to climb up to the top, it is very pleasant to stay there, for the higher we go the healthier we find it, there is clearer light, purer air, and an extensive view.

We may build up our spiritual life in the same way, for if our earthly means are small and we find it difficult to get our daily bread, yet our souls may mount in heavenly meditation, relying that Providence who fed many thousands with five loaves will enable our one loaf to be enough for all our needs. So, too, He that healed the sick, cured the lame, made the blind to see, the deaf to hear, and the dumb to speak, is nearer to us when we have climbed the mountain by prayer and praise; and He will raise us from our sick bed in His own good time to serve Him with all our members. "Higher, my soul, always higher," should be our motto, as our wishes and hopes stretch up to the mercy seat of our Father who is in heaven. The lark's song is sweetest when the bird is almost lost to sight in the blue vault above us, where he is pouring out those glorious hymns of which we only catch the first and last cadences; our prayers will be purer as we rise above the paltry wants of life and the outcries of temptation, till our souls seem to lose sight of earth and shoot like fire upward to their centre, which is above, and not below. But to return to our first thought. In buildings of wood, or stone, or brick, the top rooms are commonly the most empty, but with regard to our minds the higher we mount up the better they will be filled. Love, Hope, Patience are the valuables which furnish and ornament the heart which longs after and aspires to God. Then as we go on higher and higher, "Let perseverance to death be our uppermost chamber, the roof of which grace is the pavement of glory."

### Appointments.

Miss Anna Menon, recently appointed to Wolverhampton Infirmary, tells us that she was trained at the London Hospital as well as at Whitechapel Union Infirmary.

Ramsay Hospital, Naini Tal.—Miss A. S. Wildman has been appointed Lady Superintendent of this hospital. Miss Wilderman has been in charge of the women's medical wards at Addenbrooke's Hospital, Cambridge.

Longton Cottage Hospital.—Miss H. Lawrence has been appointed Matron of this hospital. She was trained at Addenbrooke's, Cambridge, and St. Mary's, Paddington. Miss Lawrence was Head Nurse at Addenbrooke's Hospital, Sister at Winchester, Matron of Dorset Cottage Hospital, and Night Superintendent at Salisbury Infirmary.

Children's Hospital, Bradford.—Miss Harriet Green has been appointed Matron at this hospital. She was trained at Guy's Hospital, and was afterwards Sister at the Glasgow Children's Hospital, and then Assistant Matron for two years. Miss Green was subsequently Maton of the Trinity Convalescent Home at Eaglesham, and we wish her every success in her new work.

District Infirmary, Ashton-under-Lyne.—Miss Alice Chapman has been appointed Matron of the District Infirmary, Ashton-under-Lyne. Miss Chapman was trained at Manchester Royal Infirmary, was afterwards Head Nurse at Oldham Infirmary, Night Superintendent at Victoria Hospital, Burnley, and Matron at Rochdale Infirmary. We wish her every success in present appointment.

### The Muses' Looking Glass.

### THE LIFE AND WORK OF JOHN RUSKIN.*

It is not often that the life of any man is written during his lifetime with the fulness and candour which characterise these volumes. The biographer has had rich materials at his disposal, and has shown remarkable tact and judgment in selecting only what was calculated to throw light on the growth and development of the great personality he has aimed at unfolding to the public. Writing avowedly as a disciple and admirer, Mr. Collingwood has yet slurred over none of the inconsistencies in teaching and impetuosity in action which have from time to time made Mr. Ruskin an easy mark for the most short-sighted critic. Mr. Ruskin's work, always intensely individual, acts as a reflection of himself in each period; his life and his work are inextricably bound up together, and neither can be adequately discussed without the other. In treating them for the first time as a whole, presenting both the grandeur and the defects of some great monument of nature, Mr. Collingwood has rendered a real service to literature. Mr. Ruskin has lived to see the immature conclusions of his youth revered as classics among all the English speaking races, while the ripe utterances of his later years meet a welcome chiefly from a somewhat narrow clique of "Ruskinians." It has been the part of the biographer to show that his message and life purpose has in effect been one since the days of his baby sermon: "People be dood. If you are dood, Dod will love you. If you are not dood, Dod will not love you. People be dood," to the almost equally simple address to the Coniston Sunday-school children in 1881, "We must look to the Saviour to deliver us from our sin. It is right we should be punished for the sins which we have done; but God loves us, and wishes to be kind to us, and to help us, that we may not wilfully sin." Between those utterances lies a stormy life time of doubt and pain and failure, out of which emerging the prophet may discern himself amidst a new generation for whom he has won a purified canon of taste, new perceptions of the beautiful and recovered ideals, and seeing of the travail of his soul shall be satisfied.

Born in London near Brunswick Square on the 8th of February, 1819, John Ruskin was not endowed by nature with a constitution particularly well adapted to the long life of incessant energy which lay before him. His early education was a good deal interrupted by ill health, and at twenty, after a disappointment in love, symptoms of consumption appeared, from which, complicated by a spinal affection, he suffered during all the early years of his manhood. The strain on the over sensitive nervous system provoking in later life acute attacks of mental illness, made him liable to periods of despondency in which his only refuge was his habit of ceaseless industry. His ways of doctoring himself were as might be expected unique. "Though one of his best friends is a physician, and another a surgeon, he usually prefers to be his own doctor as long as he can, and believes more in diet and exercise than in medicine. The local practitioner who attended him (this was in 1870, during an attack of internal inflammation) still tells how he refused remedies, and in the height of the disease asked what would be worst for him. I understood at Matlock that the answer was 'sherry;' Mr. Ruskin himself says it was beef; anyhow, he took it, and to everybody's surprise, recovered."

Mr. Collingwood deals fully with the common accusation of insanity freely laid to Mr. Ruskin's charge by the public at every unexpected development in his opinions. "He had been accustomed to hear himself called mad—the defence of Turner was thought by the dilettanti of the time to be only possible to a lunatic; the author of 'Stones of Venice' was insane in the eyes of his critic, the architect; it was seriously whispered when he wrote on Political Economy that Ruskin was out of his mind, and so on." And whimsical enterprises such as the sweeping of the streets in St. Giles's, and the road-mending of his disciples at Oxford, seemed to certain prosaic people to place his insanity beyond question. Moreover, "the brain fever of '78, so difficult to explain to his public (it followed a series of cruel disappointments and vexations), made it appear that the common reproach might after all be coming true. The recurrence of a similar illness in 1881 made it still more to be feared. It seemed as though his life's work was to be invalidated by his age's failure." The question resolves itself to this "Is he more or less logical, rational, coherent in mental development than other men to whom we listen and in whom we trust for opinion and advice?" And Mr. Collingwood's answer, based on many years of close intimacy with Mr. Ruskin and knowledge of every varying manifestion of his passionate convictions is summed up as follows: "The more I study his life the more I see that his work is not irresponsible and eccentric. The careful student should be able to trace his genius down to the end in continuous and rational progress. Passing over defined intervals of mental disease, and allowing for vehemence of expression—partly characteristic, partly the temporary effect of the penumbra of the storm cloud—his mental development, I make bold to say, is normal and logical throughout his life. And, I believe, that when his work is looked back upon as a whole, with proper understanding of its environment, and with full knowledge of its circumstances, the common reproach of insanity made against each new manifestation of his mind will then be scorned as an exploded prejudice."

There are few more interesting passages in these volumes than the occasional record of some such "new manifestation of his mind." In 1845 we find him "breaking the Sabbath" for the first time in his life by climbing a hill after church! "That was the first shot fired in a war, in one of the strangest and saddest wars between conscience and reason that biography records." The passage from the scepticism of his middle period which took place at Venice thirty years later is thus related:

"Christmas Day (1876) was a crisis in his life. He was attacked by illness; severe pain, followed by a dreamy state in which the vividly realized presence of St. Ursula, mingled with memories of his dead lady, whose "spirit" had been shown him just a year before by a "medium" met at a country house. Since then he had watched eagerly for evidences of another life, and the sense of its conceivability grew upon him in spite of the doubts which he had entertained of the immortality of the soul. At last, after a year's earnest desire for some such assurance, it seemed to come to him. What others call coincidences, and accidents and states of mind, flashed for him into importance; times and seasons, names and symbols, took a vivid meaning. His intense despondency changed for a while into a singular happiness—it seemed a renewed health and strength; and instead of despair, he rejoiced in the conviction of guarding providences and helpful influences. He found now new excellences in the early Christian painting; he depreciated Turner and Tintoret, and denounced the frivolous art of the day. He searched the Bible more diligently than ever for its hidden meanings—and in proportion as he felt its inspiration, he recoiled from the conclusions of modern science, and wrapped the prophet's mantle more closely round him, as he denounced with growing fervour the crimes of our unbelieving age." K.

* "The Life and Work of John Ruskin." By W. G. Collingwood. (Methuen and Co.)

### Notes and Queries.

### Queries.

(164) *America.*—Will you kindly tell me of a good American nursing paper?—*Nurse J. M.*

(165) *Training.*—Where can I find particulars of English training schools?—*Miss Grace.*

### Answers.

(164) *America* (*Nurse J. M.*)—"The Trained Nurse" is a good magazine, issued monthly by the Lakeside Publishing Company, 16, Vesey Street, New York, price 20 cents.

(165) *Training* (*Miss Grace*).—"How to Become a Nurse," by Honnor Morten, price 2s. 6d., at Scientific Press, 428, Strand.

### The Book World for Women and Nurses.

[We invite Correspondence, Criticism, Enquiries, and Notes on Books likely to interest Women and Nurses. Address, Editor, The Hospital (Nurses' Book World), 428, Strand, W.C.]

**The English Baby in India and How to Treat it.** By Mrs. Howard Kingscote. (J. and A. Churchill.)

This little book, price 2s. 6d., is prefaced by remarks from Surgeon-Captain R. H. Firth, A.M.S., who truly states that "there is need of a better knowledge concerning the management of English babies in India, especially among the wives of the humbler officials of both the civil and military services. The authoress, however, gives a list "of the usual layette of a new-born infant," which, although desirable, is far more complete than the wives of the humbler classes mentioned above could as a very general rule afford to procure. Then we have many cautions about Ayahs and Aymahs, the authoress having apparently met with more than unusually inferior specimens of such domestics; and whom very few of the humbler classes can maintain. Chapter IV. is devoted to diet of infants, and the mother is recommended to try and nurse the child herself. But it may be broadly stated that, as the result of residence in India, the majority of European women are physically unable to nurse after the second or third confinement. With every desire to fulfil such duties they find their strength unequal to the strain. It would have been well, therefore, if something had been said on this point, and the symptoms of, and results of, over-nursing been detailed. The advantages and disadvantages of Swiss milk, and various other diets for infants are very fairly stated, and the advice given may be safely followed. Mrs. Kingscote says, "What suits one child may not suit another," and that if the child appears well the advice so frequently tendered by friends of something to suit better should not be followed. As regards vaccination, the authoress says, "If possible have the vaccination done from a white child." Now this is simply a sentimental feeling; for the operation is quite as likely to be successful and harmless from an Indian as from an European child. The question resolves itself into whether the child from which vaccine lymph is taken is a healthy child or not. Skin colour makes no difference whatever. Other chapters are devoted to sleep, air and exercise, and teething. We quote one line of good advice, "Never give a child a sleeping draught however troublesome it is." But we do not agree with the following: "A very prevalent habit in India is to send the child out in the early morning before or without its bath." Mrs. Kingscote thinks the child should be bathed, or at least sponged before it is dressed. But this would entail much delay, or rising half an hour or so earlier. Already it is necessary to rise with the first glimpse of dawn so that an airing may be obtained before the sun grows too hot, and bathing is best delayed until the return of the child to the house. With reference to teething the authoress tells us that "her experience goes to show that teething is, as a rule, much easier, more rapid, and less painful in India than in England." This may be questioned. But the next sentence may be fully accepted, "Many little ailments which attend teething

### THE BOOK WORLD FOR WOMEN AND NURSES—*continued.*

need more attention than at home." This observation is specially applicable to diarrhœa or bowel complaint. Chapter IX is headed "Minor Ailments," and included under this denomination are convulsions, croup, congestion of the liver, infantile diarrhœa, and various other diseases which we certainly do not regard as minors! In another chapter cholera, fever of different kinds, including scarlet and typhoid, and other affections are treated of. At the end of the volume are a number of prescriptions and a recommendation of "two invaluable foods, Welford's and Frame Food." Now some part of Mrs. Howard Kingscote's book is very good. Most of the advice relating to the management and dieting of infants may be read and generally acted upon with advantage. So also with regard to what is said to the young mother. But when Mrs. Kingscote writes of the ailments mentioned above, gives prescriptions and practically assumes the rôle of a medical man through at least half the 184 pages of which the volume consists, Mrs. Kingscote does, in our opinion, attempt too much, especially as there are other books in which the maladies of children in India have been treated of by medical authors.

The New Border Tales. By Sir George Douglas, Bart. (Walter Scott.) Price 3s. 6d.

One closes this volume of tales with a feeling that it is well our lines are not cast amongst the very unpleasant characters to whom the author introduces us. The stories are no doubt faithful sketches of Border life in days not long gone by, many, if not all, founded on traditions handed down among the peasantry; but we could wish Sir George Douglas had found some of a less dismal nature. Murder and treachery form the theme of nearly all, yet the stories are well written, and we are interested in spite of ourselves. "The Brither Stanes" may be taken as an instance. The "Stanes" are two upright blocks of whinstone, which commemorate the following tragedy. Walter and Ninian Cranstoun are the twin sons of an unpopular laird, who himself was foully murdered whilst returning from St. Boswell's Fair. Never the best of friends, the boys fight on the very day of their father's death, Walter, the younger, receiving a wound on his temple, of which he carries the scar for the rest of his life. The boys grow up to manhood, and one day, in a somewhat unfriendly spirit, they engage in a wrestling match. They fall together, and the little dirk worn by Walter escapes from its sheath, and pierces Ninian's side. Overcome with horror, Walter strives to staunch the flow of blood, but to no avail, and, believing his brother dead, he flies from his home. But Ninian recovers. Years pass, and no news is received of the younger brother. Ninian, as he grows older, becomes morose and violent. Riding home one day he passes a little inn, where he hopes for refreshment, but finding the place deserted, he breaks the window, enters, and soon makes short work of the landlord's Hollands. While thus engaged a stranger arrives, apparently some soldier of fortune, and the two drink together. Presently, fashioning some rough dice, they proceed to play. Discovering that the unknown soldier is cheating, a quarrel ensues, in the course of which the stranger falls, mortally wounded. Then Ninian recognises his long-lost brother, and, mounting his horse, flies from the scene. In the morning the horse is found with the lifeless body of the rider dragged behind him from the stirrup. And the spot where stand the stones raised by the neighbours in memory of the tragedy, to this day bears the name of "Brotherston." The book is well illustrated.

